# Life lessons after classes: investigating the influence of an afterschool sport program on adolescents’ life skills development

**DOI:** 10.1080/17482631.2017.1307060

**Published:** 2017-04-03

**Authors:** Okseon Lee, Mirim Park, Kyunghwan Jang, Yongnam Park

**Affiliations:** ^a^ Department of Physical Education, Seoul National University, Seoul, Korea

**Keywords:** Life skills, afterschool, sport program, youth development

## Abstract

The purpose of this study was to investigate the influence of an afterschool sport program on adolescents’ life skills development and to identify which characteristics of the program would have an influence on their life skills acquisition. The participants were six children (4 boys, 2 girls) who participated in a 12-week afterschool program implemented in two elementary schools, as well as the two program instructors who implemented the afterschool sport program. Data were collected from individual interviews with program participants and instructors. The inductive analysis of data revealed four categories of life skills developed through program participation: (1) playing well and being more active, (2) connecting well and having better social skills, (3) coping well and becoming a better problem solver, and (4) dreaming well and having a better sense of purpose. Regarding the characteristics of the program that influenced life skills development, three themes emerged: (1) having a clear goal and building consensus with stakeholders, (2) establishing a firm yet flexible structure, and (3) instructors’ use of effective strategies for teaching life skills.

## Introduction

Afterschool hours have been considered as both risk and opportunity for the health and wellbeing of adolescents. The rise of youth risk behaviors such as violence, smoking, drinking, and substance use (Dwyer et al., 1990; Kahne et al., 2001) associated with unproductive use of non-discretionary time has heightened researchers’ attention to the importance of using afterschool hours as a unique opportunity for providing skill building and enriching experiences for adolescents. Given that much literature supports the educational value of afterschool programs for healthy development of adolescents (Durlak & Weissberg, 2007; Shernoff, 2010), afterschool programs have been considered as another school within school system that extends and enriches regular curricular activities. In Korea, about 61.8% of elementary school students are participating in at least one afterschool program, and among these, sport and physical activity programs are the most popular, with 4331 school-based afterschool programs being offered nationwide (Korean Ministry of Education, 2015).

Although there has been a drastic increase in the number of afterschool sport programs, there has been a lack of effort to enhance the quality of the programs. Afterschool programs have come to cover a relatively broad spectrum of goals, such as providing child care, complementing regular curricular activities, and helping adolescents stay away from problem behaviors (Korea Institute for Curriculum and Evaluation, 2012; Noam, Biancarosa, & Dechausay, 2003) by encompassing the diverse needs of the school and society. Despite the drastic growth of the number and expansion of goals, in Korea there has been no framework that has provided guidelines for “what” and “how” to teach in afterschool programs that develop well-rounded adolescents (Lee, 2015).

Given the broad spectrum of expected goals of afterschool programs, the positive youth development (PYD) approach can be a framework that guides the development of adolescents in sport-based afterschool programs. The PYD is a strength-based approach, focusing on strengths, assets, and competencies rather than focusing on reducing deficits or problem behavior (Lerner, 2002). Damon (2004) highlights the importance of strength building rather than risks and deficits by stating that PYD “emphasizes the manifest potentialities rather than the supposed incapacities of young people” (p. 15). By focusing on strengths and assets building, the PYD approach emphasizes that youth development should be targeted for *all youth* rather than focusing on the ones who have immediate needs or problems (Benson, Scales, Hamilton, & Sesma, 2006).

In order to capitalize on afterschool sport programs as a context for PYD, the program should have a specific focus and structured opportunity for skill-building in order to develop appropriate relationships with adults (Roth & Brooks-Gunn, 2003). Regarding what skills or knowledge adolescents should develop, the life skills development approach has been widely accepted in youth development research and practice. The World Health Organization (WHO, 1999) defines life skills as specific personal, social, interpersonal, cognitive, and affective skills that are needed in order to deal with the demands and needs of life. In a similar way, Danish and his colleagues (2004) define life skills “those skills that enable individual succeed in the different environment in which they live, such as school, home, and in their neighborhood” (p. 40). Therefore, transferring life skills learned in a sport context to their daily life contexts is one of the critical defining characteristics of developing life skills.

Given that sport and physical activity programs are very attractive and a “hook” for the majority of adolescents (Hartmann, 2003), they also work as a moral laboratory to practice decision-making and problem-solving skills, as well as teamwork and cooperation (Clifford & Feezell, 2009). Many studies have explored the possibility of teaching life skills in the context of sport and physical activity programs. The development of life skills has been examined within the context of elite sport because life skills development has been considered an approach to offset the negative cost of pursuing performance excellence, (e.g., eating disorders, poor career planning) while still achieving personal excellence (Miller & Kerr, 2002). Studies have shown that athletes learned life skills such as goal setting, motivation, positive skills, and communication skills in an elite sport context. Because of this, teaching both life and performance skills was possible in sport programs (Jones, Lavallee, & Tod, 2011).

Another major context for life skills development was seen in programs geared towards the underserved youth population. Given that underserved youths have limited resources and access to programs that promote positive development, the potential of using sport to teach life skills development has been one of the major foci of the research. These studies showed that sport programs that had been provided to at-risk youth helped to enhance their life skills, career exploration skills (Walsh, 2008), youth leadership (Martinek, Schilling, & Hellison, 2006), and school engagement (Martinek, McLaughlin, & Schilling, 1999).

The studies on the impact of sport-based programs on adolescents’ life skills development, however, have mainly focused on either at-risk or elite student athletes, rather than focusing on the vast majority of adolescents who participate in sport as a structured leisure activity in afterschool program settings. Given that the PYD approach focuses on *all* youth rather than *some* youth who have urgent needs, more studies should be conducted on adolescents who participate in sport as a structured leisure activity. It adds more importance to the Korean context because there has been a drastic increase of afterschool sport programs since the late-2000s (Lee, 2015), yet few studies have examined the impact of afterschool sport programs in terms of adolescents’ life skills development. In addition to this, the research should also focus on the characteristics of the program and how they influence the development of the children’s life skills. Life skills development requires intentional and structured opportunities. Although some studies have examined the positive physical, social, and psychological outcomes of afterschool sport participation (Kahne et al., 2001; Mahoney, Lord, & Carryl, 2005), few studies have focused on what characteristics of programs have influenced these positive outcomes. In the end, the lack of information treats program characteristics as if they were a black box (Durlak & Weissberg, 2007; Shernoff, 2010).

Consequently, the purpose of the study was to explore what specific life skills adolescents develop through the participation in a sport-based afterschool program. The study also sought to identity the characteristics of the program that influenced the adolescents’ life skills development. The findings of this study will provide insights into the design and implementation of sport-based afterschool programs by identifying “what” and “how” life skills are developed in an afterschool sport program setting.

## Methods

This study was conducted as a qualitative program evaluation study, examining program processes and outcomes (Patton, 2002). As for program processes, this study focused on identifying program characteristics influencing children’s life skills development. As for program outcomes, the influences of the afterschool program on children’s life skills development were examined. The program participants and two instructors were included not only as data sources but also as important stakeholders who might enhance the content and structure of the program by providing specific and relevant information.

### Context and setting

A 12-week afterschool sport program called Playing for Fun and Life was designed to teach both life skills and sport skills to adolescents. Given that soccer was one of the most popular sports among Korean adolescents, the program selected soccer as an avenue and context to teach the four areas of life skills: individual player skills (e.g., participation), team player skills (e.g., playing *with* and *for* others), competing player skills (e.g., playing *against* all odds), and thriving player skills (e.g., setting long-term goals). The first phase of the program (sessions 1–3) focused on developing life skills for individual students (e.g., self-confidence, doing one’s best, self-management), and then it moved toward the second phase of the program (sessions 4–6), which focused on team-related life skills (e.g., teamwork, communication). After sessions 1–6 were completed, participants learned how to handle pressure, stress, and conflicts in the third phase of the program (sessions 7–9), and completed the program by exploring future plans and setting short and long-term goals (sessions 10–12).

The program was implemented in two elementary schools by two different instructors. In order to ensure consistency of content and methods of teaching life skills, a program manual was provided to the two instructors and both of the instructors participated in the three sessions of staff training provided by the university regarding the goals of the program, specific teaching strategies for life skills development, and management and organization of the afterschool sport program.

### Ethical considerations

The study was approved by the first author’s University Institutional Review Board. Informed consent was obtained from the children’s parents and program instructors. In addition, children were given their own consent for the study. The provided consent included information on: (1) overview and purpose of the study, (2) procedure of data collection (e.g., frequency, length, choice of interview place, prior notice of recording), (3) risks and benefits, (4) assurance of confidentiality (e.g., using pseudonyms), (5) right to withdraw from the study at any time, and (6) contact details of the primary investigator. The children were also provided with the same information using a child-friendly verbal explanation. Children were informed as to why we were doing this study, what were the positives or negatives that might occur during the study, who would be told the things we learned about them and their peers, what they should do if they changed their mind about participating in the study, and who they should contact if they had any questions. When children agreed to participate, they provided us with their own consent.

### Participants

A total of six children (four boys, two girls), three from each school, agreed to participate in the study. The average age of the participants was 11.5 years old, and they were fourth and fifth graders in elementary school. In addition, the two program instructors also participated in the study. One of the instructors, Mr. Lee, was a former soccer player and had worked as part of the staff for a youth sport program for life skills development with student athletes as well as a community-based sport mentoring program. The other instructor, Mr. Kim, was an elementary school teacher who had seven years of teaching experience in elementary school and three years of afterschool sport program teaching experience. The two instructors strongly agreed with implementing a sport-based life skills development program in an afterschool setting.

### Data collection

Data were collected from individual interviews with children and program instructors and observations of the afterschool program sessions. Children were interviewed twice, before and after their participation in the program. The pre-implementation interview focused on the children’s afterschool activities, motivations to participate in the program, and key personal details. The post-interview focused on children’s life skills outcomes and the factors that influenced their life skills development. The questions for the pre-and post-implementation interviews are presented in Table 1.

**Table 1.** Interview questions for the children.

Phases	Questions
Pre-implementation	What do you usually do during after school hours?With whom do you usually hang out?If you were not in the afterschool program, what would you choose to do during afterschool hours? What made you participate in the program? What are your expectations for the program?What you do want to learn or do in the program? Tell me three things about yourself.Tell me about your closest friend.Tell me about your dreams.Tell me about your concerns or stresses in school or at home.
Post-implementation	What did you do or learn in the program?What was the most important thing you learned in the program?Have you ever learned similar things in class or at home?Were there any differences between things you learned here or other places? Have you tried what you learned in the program in school or at home?If you answered yes, did it work?How did it make you feel?Has your behavior changed following your participation in the program? How did the program instructor help you learn?What did he do or say? Can you give me an example?

The two program instructors were also interviewed pre- and post-implementation of the program. The pre-implementation questions included: (1) specific life skills needs for adolescents, (2) instructors’ philosophy of the program, (3) instructors’ perceived competency in implementing the program, and (4) expected outcomes for the adolescent through program participation. The post-implementation questions focused on identifying: (1) any critical incidents that happened during program implementation, (2) changes of adolescents’ behaviors and attitudes through program participation, and (3) specific strategies used by instructors for teaching life skills. All interviews were recorded on a digital voice recorder and transcribed verbatim.

### Data analysis

The researchers read the interview transcripts repeatedly and made marginal notes to facilitate the initial interpretation of the data. After the initial understanding of the data had been reached, the researchers segmented relevant parts of the data for coding (Creswell, 2013). After conducting the initial coding, the researchers then compared the initial codes to one another and grouped similar codes into sub-categories. The sub-categories were also compared with one another, and were grouped into higher levels of categories when similarities were found. Then the researchers identified emerging themes and developed explanations by considering relationships among the themes.

In order to enhance the trustworthiness of data, the researchers used  research participants  to check the participants’ intended meanings, which were represented appropriately in the process of the data analysis and interpretation. In addition, peer debriefing with two external researchers who had multiple years of experience in qualitative research was used to enhance the trustworthiness of the data (Creswell, 2013).

## Findings

The findings of this study are presented based on the two research questions: (1) types of life skills developed in an afterschool sport program and (2) characteristics of the program that influenced adolescents’ life skills development. All names used in the Findings section are pseudonyms to protect the identity of the participants.

## Impact of the afterschool program on adolescents’ life skills development

The findings revealed that the afterschool program facilitated the adolescents’ life skills development in the following four areas: playing well, connecting well, coping well, and dreaming well.

### Playing well

The first theme, *playing well*, refers to skills that deal with being more physically active, improving levels of sport skills, and having fun and enjoying physical activities and sport. Given that sport-based programs emphasize learning about and learning through sport, participants’ commitment to sport is a prerequisite for developing other areas of life skills. The findings revealed that the program changed participants’ use of afterschool hours in more physically active ways by providing structured alternative activities.

For example, when asked about his typical afterschool activity, a fifth-grade boy, Yubin, said, “I play computer games or play games with my smartphone because I don’t have anything special to do.” Through participating in the program, the inactive and unproductive use of his afterschool hours has been substituted with sport-related activities. In addition, participants perceived that their skill level had improved and they came to enjoy sport because their enhanced skill levels gave them better chances of playing sport with others. When asked what she learned in the program, a fourth grade girl, Chaemin said:

I would say enjoyment is the most important thing. I couldn’t enjoy playing soccer because I was not good at it. I once mistakenly shot the ball into our own team’s goal, and my friends blamed me a lot for that. But here I learned skills like dribbling, passing, and shooting, and I found that it was really fun to play soccer with friends because I can play better than before.

The acquisition of “playing well” skills was facilitated by creating synergies between sport skills and life skills. For example, a fifth-grade boy, JaeYoung, commented on the benefit of learning life skills integrated activities: “Here I was more into soccer and had more fun because each week we have a theme to learn, like a life skill, and it gave me the feeling that I was learning something important besides soccer skills.” In addition, participants had a better chance of practice within a more positive program atmosphere due to the fact that the program emphasized life skills such as respect, teamwork, and fair play.

The program instructors emphasized the importance of feeling deep enjoyment and staying focused on the activities as a way to teach “playing well” skills. At first, students were easily distracted from the program and they checked their smartphones even during the game. The program instructors asked the students to recite the goal of the task and verbally repeat the cues of skills in order to enhance the concentration of the participants. A participant, Donghan, reported that these techniques not only helped him to stay focused on the task but they also helped him transfer these skills into a different, but similar context:

Before I came to the program I just played the game without thinking. When the teacher asked me to meditate with my eyes closed, I used to chat with others with my eyes open. After I learned how to stay focused, I kept repeating what I should do as I learned here, and it worked even during PE classes.

Developing the skills for playing well was also related to the contextual characteristics of afterschool programs. For instance, the instructors would say, “We don’t have a test or exam here” and “We have more freedom here than in school PE.” Unlike regular curricular activities, the afterschool program had more flexibility in terms of structure and choice of content. These contextual characteristics helped the students be more committed to the program and be more motivated to learn and enjoy the program.

### Connecting well

The second theme, *connecting well*, refers to the skills related to working *with* and working *for* other people. Given that most of sport activities require teamwork and intensive interaction with other people, sport worked as a good medium to teach life skills. For example, Ingang explained how he learned the importance of caring for others when he learned passing skills:

When we practiced passing skills, Mr. Lee said, “Passing is the act of caring. You have to think where your teammate is and show that you care for him so that he can receive it well.” If I give a good pass, then my friend will give me back a good pass. It is same when we talk to other people. We have to think about other people’s feelings before we say something. When we pass along some nice words to others, then they will return nice words to us. I think it is the key to getting along with others.

Participants also reported that they learned the importance of teamwork and cooperation through soccer activities. It is achieved through emphasizing the value of teamwork and cooperation rather than competition and winning. Regarding this, JaeYoung, a fifth grade boy, commented:

I used to play just to win, but here we were supposed to help others and pass the ball to others before shooting for a goal. It made me feel closer to my friends and I learned the importance of working together.

In order to connect well with others, participants also learned how to communicate with others through a structured game play provided by the instructor. For example, Mr. Kim designed a game called silent soccer so that participants could experience the importance of active listening and respecting other people’s feelings and thoughts. Commenting on how she learned communication skills, a fourth-grade girl, JS said, “When we played silent soccer, it was really frustrating because we couldn’t talk. We had to focus on other people’s gestures and signals and become more sensitive to other people’s feelings to work as a team.”

The skills of connecting well with others have been transferred to participants’ other areas of life such as school. For example, Ingang reported how he found the applicability of life skills learned in the sport context to the classroom as follows:

I learned how to get along with others here. I felt like, “Aha! This is the way to get along with others.” I had to listen to others, show that I cared for them, and be a good example. When I became a class president, I took the role just like playing a soccer game in the field. I closely observed my friends, listened to them, and did something when it was needed before being asked to do. When I acted like that, they liked me and happily followed me.

Given that participants spent their afterschool hours in individualistic activities such as watching TV or playing computer games before they joined the program, the development of connection skills has crucial importance in adolescents’ lives. The participants’ acquisition of connection skills was related to (1) the characteristics of afterschool programs, which provided ample opportunities to interact with participants from diverse age groups, and (2) the characteristics of a team sport and the instructors’ intentional use of specific strategies, such as structured game play to develop life skills.

### Coping well

The third theme, *coping well*, refers to the life skill required to deal with stress, problems, or conflict in a constructive way. The pre-implementation interview with participants showed that participants’ coping with stress was rather destructive and lacked specific strategies. For example, when asked how he responded to stressful situations, Yubin said, “I yelled at others and showed my anger so that they wouldn’t talk to me anymore.” The rest of the participants reported that they responded to stress by crying, punching pillows, or eating sweets.

However, participants reported that they came to have better coping skills through afterschool sport program participation. The program not only provided specific tools but also provided an alternative context and activities to deal with stress and problems. For example, when they were stressed with an exam or test, participants applied specific coping strategies such as deep breathing or self-talk, which were both learned in the program, to handle their stress. In addition, they used sport as an alternative activity to release stress rather than eating or playing computer games. Regarding how she changed her approach in dealing with stress, Chaemin commented:

When we practiced soccer shooting, Mr. Kim asked us to shout, “Stress, kick out!” whenever we kicked the ball. It made me feel better and I decided to play sport when I get stressed rather than playing a computer game. I also learned to use deep breathing and say a positive thing to myself, such as “All is well” when I was stressed with a math exam.

Participants also reported that they learned how to respond to their mistakes or the mistakes of others in a more constructive way. Given that sport experience provided vivid examples of dealing with mistakes and failures, the instructors used these opportunities to teach how to encourage others as well to be resilient in the face of their own failures or mistakes. Regarding how he learned and applied coping skills to a similar context, Ingang said:

When people made mistakes, I used to blame others for not doing well, and sometimes it ended up with a fight. Mr. Lee taught us how to build up others instead of blaming them. He said, “Build up others, don’t blame others.” We repeatedly practiced, “It’s OK. You can do better next time” whenever someone made a mistake. I used it in my PE class when a friend ran to the third base instead of the second base. Although he made three outs, I said, “It’s all right. It’s just a game. You will do better next time.” I found that it made me feel better and my friend also thanked me for my encouragement. When I made a mistake, I said the same thing to myself and it made me feel more comfortable.

In addition, participants developed coping skills, which were related to dealing with winning or losing in competition. Initially, they equated winning with success and losing with failure. However, their perceptions have now changed because they have been exposed to the idea of learning how to be a good loser and a humble winner. Regarding how he changed his attitude to deal with win and loss in the game, Donghan said, “I always wanted to win because winning tastes sweet and losing tastes bitter. But Mr. Lee said we should put forth our best effort and should not lose heart because there will always be other games.”

In order to develop adolescents’ coping skills, the program instructors put a strong emphasis on life skill development rather than just teaching how to play sport well. Regarding the importance of coping skills, Mr. Lee said, “For these children, learning about how to resolve conflicts or cope with difficult situations is more important than winning the game because they are not elite athletes. They need life lessons, not victories.” The adolescents’ acquisition of coping skills was possible when the instructor had the clear philosophy and intention to teach life skills in the sport program.

### Dreaming well

The fourth theme, *dreaming well*, refers to skills that are related to exploring future lives and setting specific goals to achieve a future dream. The pre-implementation interview revealed that participants had a very vague notion of a future image of their lives. For example, when asked to list a few career options, Donghan said, “I wanted to be a police officer, but now I want to be a president.…I want to be in the highest position and make lots of money.” Yubin’s description of his future dream was not much different: “I want to be a pro-gamer because it looks cool to be a good gamer and they make lots of money.” These comments showed that participants did not have the structured opportunities and appropriate information needed to explore their future careers.

Although it was a basic level of exploration, participants reported that they had an opportunity to think more specifically by examining their own strengths and weaknesses. For example, Chaemin reported:

I only thought that I should study hard to achieve a dream. But here we were asked to think about our strengths and weakness, and Mr. Kim said that we need to turn our weakness into a strength in order to achieve a dream.

Another participant, Ingang, also addressed the importance of specific efforts to explore self and future careers by stating that, “I learned that preparing for a job means more than studying hard. I should know what I do well, what I like, and what kind of jobs are out there.”

Unlike elite sport programs, which pressure students to win in the competition, the afterschool sport program provided an opportunity for the participants to share their lives and future dreams, which facilitated career exploration skills. Given that participants had limited social capital to explore future lives, the afterschool sport program instructors played an important role in helping them transform their vague and simple notions of future aspirations into more specific life skills. Regarding this Mr. Kim said:

Because the goal of this program is to develop life skills rather than win at the competition, I was able to share with them their future dreams, what they like, and what they can do well. I think asking them to write down their dreams, change the dreams into specific goals, and take time to think about what they should do, on a daily basis, was helpful to them.

## Characteristics of program influencing life skills development

Three themes emerged regarding the characteristics of the program that influenced adolescents’ life skills development: having a clear goal and building consensus with stakeholders, establishing firm yet flexible structure, and instructors’ use of specific teaching strategies.

### Having a clear goal and building a consensus with stakeholders

The two program instructors commonly reported that they had a clear goal in teaching life skills in an afterschool sport program. For example, Mr. Lee, expressed his opinion that afterschool programs should be both educational and life skills oriented so that it can have transferrable and sustainable impacts on adolescents’ future lives. Explaining his teaching philosophy in the afterschool program, Mr. Lee revealed:

Although it is offered during afterschool hours, afterschool sport programs are definitely an educational activity. I have a firm belief that students should learn life skills because those are what they need in their lives. They need skills for promoting health, having empathy toward their friends, and getting along with others. I focus on long-term goals so that they can obtain and use life skills in their future lives.

Another instructor, Mr. Kim, reported that he clearly communicated his intention and program philosophy with parents and adolescents in order to build a consensus with them. The program instructor perceived that teaching life skills should be intentional and also require mutual understanding both from parents and adolescents in order to obtain appropriate support. Commenting on how he built a consensus with stakeholders, Mr. Kim said:

When I recruit members, I explained the goals and intention of the program. I want to recruit members who understand my philosophy. Parents showed more interest in the program when I told them I will focus on life skills as well as sport skills. Although they didn’t know exactly what life skills were, they seemed to understand the importance of developing positive qualities in addition to sport skills. When parents understand and have trust in the program, they tend to be more supportive of what we are doing here.

In addition, the program instructors constantly emphasized the goal of the program so that participants internalized the value of learning life skills. The instructors admitted that implementing the life skills oriented program took time because participants were accustomed to traditional sport programs organized around game play and skill practice. Regarding this, Mr. Lee said, “At first, children wanted to play games rather than learn life skills. Although it was difficult, I constantly emphasized life skills. Eventually, they took pride in themselves because they were learning these life skills.”

When program goals and philosophy were clearly communicated, participants reached a consensus with program instructors. For example, when asked what was the most important thing learned in the program, a fifth-grade girl, Chaemin, said, “Mr. Kim used to say that when we learned only sport skills, we got only a half out of the program. He told us we have to learn life skills so that we can use them anywhere and anytime.”

### Establishing firm yet flexible structure

The two program instructors commonly addressed the importance of a systematic and structured experience for adolescents’ life skills development. To establish a program structure, the program instructors reported that they clearly communicated what was expected by setting up rules in the program. The rules provided a boundary of acceptable behavior and also helped participants establish a positive atmosphere in the program. When asked what is expected in the program, a fifth grade girl, JS, commented, “Here we can do whatever we want, but there are some rules to follow such as no trash talking, playing fair, and always encouraging and respecting others.”

The program instructors not merely established the rules but also constantly reminded them of the guidelines during the program. When participants broke the rules, the instructors revisited the rules and provided an opportunity to reflect on their behavior. Consequently, the instructors used this process as a teachable moment for teaching life skills. Regarding how he used the rules as a tool for teaching life skills, Mr. Lee said:

Because sport is very dynamic and active, children can easily get excited and lose self-control. That’s why we need a clear boundary here. When they break the rules, I usually go back to the rules of the program and asked them to think about what rules they broke and to think about the consequences of their behavior. Sitting down and having time to talk with children helped them cool down and also reminded them of the importance of the rules of the program. I think there is actually a good chance to teach life skills because some of the rules are very similar to the life skills.

Besides setting up a clear boundary, the program instructor also provided adolescents with an opportunity to take responsibility and experience a sense of autonomy. Given that this afterschool program had more flexibility in terms of content and structure, the program instructor capitalized on it to promote adolescents’ sense of autonomy. Regarding this, Mr. Kim said:

I think an afterschool sport program is the best place to learn autonomy. I can decide what to do and how to do it with my students. In this sport program, we have regular meetings and they decide who will draw lines on the playground, who will lead the warm-up exercise, and who will manage the equipment. All these are great opportunities to experience feelings of autonomy.

When adolescents were provided with a sense of autonomy, they used this opportunity to explore how they could learn life skills better. In addition, the participants developed positive reactions such as happiness, encouragement, and cooperation within the context of the autonomous program. The following comments from Ingang showed that adding choices and flexibility to program structure strengthened and deepened his life skills acquisition through self-discovery:

I learned soccer in other places but they did not give me many choices. Here I had more freedom and fun. We could choose either to practice alone or work with others. I found that I learned better when I worked with others with encouragement and cooperation. I would never know that I was good with friends if I did not have a chance like this.

The findings indicated that adolescents needed a firm structure guiding their expected behaviors as well as a flexible and autonomous atmosphere in order to take ownership of their life skills learning experiences.

### Instructors’ use of specific teaching strategies

The program instructors applied various strategies for adolescents’ life skills development. In order to teach life skills, the program instructors translated the meaning of life skills into the children’s level of language. Providing a simple yet clear definition of life skills through a one-liner turned out to be helpful. For example, when teaching the importance of caring, Mr. Lee repeatedly used the phrase, “Passing is the act of caring.” Participants were asked to repeat the phrase when they practiced soccer passing skills, and they were asked to be considerate and to think of the other peoples’ positions so that they could easily pass the ball. Using a one-liner helped participants understand the meaning of life skills and facilitated the practice of life skills in sport contexts. Regarding the use of a one-liner, Mr. Lee stated:

I usually make a short and simple phrase to make it easier to understand. For example, when I teach a life skill such as team spirit, I ask them to recite “One for all, all for one.” By reciting the phrase while they practiced, they came to understand not only the meaning of it but also when and how to use it.

In addition, the program instructors used sport metaphors to teach life skills. Given that sport settings have many similarities to real life situations, the program instructors used sport-related metaphors and stories to teach life skills. Regarding how he learned the importance of good communication skills in relation to soccer passing skills, Ingang explained:

Mr. Lee used to say, “Coming passes will be nice if gone passes were nice.” Likewise, when I give a good word to my friend, then he will return a good word to me. Mr. Lee said that relationship and communication are the same as passing skills. When I encourage other people and say something nice, they will also be nice to me.

The program instructors also used reflection as a strategy to facilitate the transfer of life skills beyond sport contexts. Reflection not only reminded them of what they learned but also facilitated participants’ thinking about how they can transfer it to real life contexts. Regarding how he used reflection to promote life skill learning Mr. Kim commented:

When I close each session, I sit down with them and have reflection time with them. I usually ask, “What did you learn today? How can you work on these in your school or home life?” Initially, children talked about sport skills, but as time went on they talked about life skills such as taking care of their health or dealing with stress. I asked them to write a short journal to record what they learned, how they learned, and how to work on it in their school or home. Filling out a simple format of a journal made it easier for them to remember what they learned and how to set goals.

Teaching these life skills to the participants was possible due to the instructors’ specific and intentional instructional strategies. The instructors adopted various strategies to clarify the meaning of life skills, help them recite and practice during sport play, and also to facilitate transference of life skills beyond a sport setting.

## Discussion

This study examined adolescents’ life skills development in a sport-based afterschool program and the characteristics of a program that influenced their life skills development. The findings of this study showed that adolescents’ participation in an afterschool sport program enhanced life skills in the four domains: playing well, connecting well, coping well, and dreaming well.

First, the afterschool sport program facilitated the adolescents’ healthy and active lifestyle by providing a structured opportunity to develop sport skills. The enhancement of sport skills and the feeling of joy through play worked as assets for the adolescents as they extended their participation beyond the program setting to maintain active lifestyles. The literature also supports the theory that participation in sport and physical activity programs promotes diverse health benefits, and provides a foundation for future engagement in active lifestyles (Beets, Beighle, Erwin, & Huberty, 2009; Fuller, Percy, Bruening, & Cotrufo, 2013). Given that there is a decreasing trend of adolescents’ participation in sport and play in non-school settings due to privatization of sport, parental concern for safety, and academic-oriented culture, providing safe and educational learning experiences through afterschool programs and developing life skills such as playing well are critically important to adolescents’ healthy development (Bailey, 2006; Choi, Park, Jo, & Lee, 2015).

Second, the findings revealed that playing sport and improving sport skills was not an ultimate outcome of the afterschool sport program, but it also worked as a vehicle to develop other life skills such as social and coping skills. It supports that sport is a viable tool to teach social skills, coping skills, and future exploration skills by providing active and dynamic contexts for practicing teamwork, cooperation, problem-solving, and goal-setting skills (Holt, Tamminen, Tink, & Black, 2009; Petitpas, Cornelius, Van Raalte, & Jones, 2005). Compared with regular curricular activities, the afterschool program provided a more suitable and flexible context to socialize with a diverse range of students and worked as a laboratory to practice diverse life skills including social and coping skills (Clifford & Feezell, 2009).

The current study also identified characteristics of programs that facilitated adolescents’ life skills development. The program instructors’ clear philosophy on the importance of life skills and their effort to build a consensus with students and parents turned out to be important. Considering that adolescents are navigating multiple contexts such as home, school, and community, clear communication of a program’s philosophy is critical in building a consensus among people in diverse developmental contexts. Additionally, this approach helps to support the adolescents’ transfer of program values to other developmental contexts (Lee & Martinek, 2013; Perkins & Noam, 2007).

Another characteristic was establishing a clear boundary yet providing a flexible structure to practice a sense of autonomy in the program. The findings are similar to the features of positive youth development such as use of appropriate structure with clear rules and expectations, use of positive social norms, and empowering youth to take responsibility and have ownership of the program (Catalano, Berglund, Ryan, Lonczak, & Hawkins, 2004). It highlights the importance of structural characteristics of the program which transforms a sport experience into a life skill learning experience and facilitates the transfer of life skills to other contexts such as home, school, and community (Petitpas et al., 2005).

Finally, the instructors’ teaching strategies, such as use of short phrases, sport-related metaphors, and reflection, turned out to be effective. The use of a one-liner and sport-related metaphors facilitated adolescents’ cognitive understanding of life skills as well as a contextualized understanding of life skills during sport play. In addition, reflection helped adolescents identity what life skills they learned and how to transfer them to other domains of life (Jones et al., 2011). These identified strategies add empirical understanding on how to teach life skills in addition to demonstration, modeling, and practice (Camiré, Trudel, & Forneris, 2012). All these strategies imply that teaching life skills is an intentional process (Danish et al., 2004) rather than a natural process.

## Conclusion

Afterschool hours have been considered as a contested context for adolescents’ development, both as a risk and opportunity. This study showed that the sport-based afterschool program can be an important context for life skills development such as playing well, connecting well, coping well, and dreaming well in adolescents’ lives. In addition, it provides information on program characteristics such as having a clear goal and building a consensus with stakeholders, establishing a firm yet flexible program structure, and use of specific and intentional strategies for life skills development. These characteristics should be considered not only for designing an afterschool sport program, but also should be emphasized for staff training so that the program staff fully capitalize on the power of sport for life skills development.

## Limitations of the study

The study has limitations in that only six children in two schools, three children in each, were interviewed. Coupled with the small number of study participants, the low age of the subjects can be seen as a limitation of the study. Because the young participants were limited in their abilities to articulate their thoughts and feelings, the transferability of this study may be somewhat restricted. In order to overcome this weakness, future studies should explore children’s life skills development by combining the observation of participants’ behaviors along with individual interviews. In addition, this study does not provide detailed explanations on how life skills acquired in sport programs were applied to different contexts such as home or school. Given that the ultimate goal of the life skills program lies in the transference of the lessons beyond the sport program setting (Danish et al., 2004), future studies should explore this issue by including diverse contributors, such as school teachers and parents, to obtain a more holistic picture of each child’s individual life skills development.
